# Phenotypic Characterization and Genome Analysis of New Broad-Spectrum Virulent Salmophage, Salmonella Phage KKP_3822, for Biocontrol of Multidrug-Resistant *Salmonella enterica* Strains

**DOI:** 10.3390/ijms252312930

**Published:** 2024-12-01

**Authors:** Michał Wójcicki, Dziyana Shymialevich, Paulina Średnicka, Paulina Emanowicz, Agnieszka Ostrowska, Hanna Cieślak, Barbara Sokołowska

**Affiliations:** 1 Department of Microbiology, Prof. Wacław Dąbrowski Institute of Agricultural and Food Biotechnology—State Research Institute, Rakowiecka 36 Str., 02-532 Warsaw, Poland; diana.szymielewicz@ibprs.pl (D.S.); paulina.srednicka@ibprs.pl (P.Ś.); paulina.emanowicz@ibprs.pl (P.E.); hanna.cieslak@ibprs.pl (H.C.); 2 Department of Nanobiotechnology, Institute of Biology, Warsaw University of Life Sciences (WULS-SGGW), Ciszewskiego 8 Str., 02-786 Warsaw, Poland; agnieszka_ostrowska@sggw.edu.pl

**Keywords:** lytic bacteriophage, *Salmonella enterica*, salmonellosis, multidrug resistance, genomic analysis, functional annotation, food biocontrol

## Abstract

*Salmonella* is one of the main foodborne pathogens. Irrational antibiotic management has led to an increase in the incidence of multidrug-resistant strains. Bacteriophages may be an alternative method of food biopreservation and contribute to reducing the number of food poisonings requiring pharmacotherapy. This study aimed to isolate a bacteriophage (phage) targeting indigenous multidrug-resistant (MDR) *Salmonella* strains, followed by their biological, morphological, and genomic characterization. In this study we isolated Salmonella phage KKP_3822, targeting MDR *Salmonella* Manchester strain KKP 1213. Salmonella phage KKP_3822 retained high activity in the temperature range from −20 °C to 40 °C and active acidity from pH 3 to 11. Temperatures of 70 °C and 80 °C and extreme pH values (2 and 12) significantly reduced the phage titer. Its activity decreased proportionally to the time of UV exposure. Genome analysis (linear dsDNA with a length of 114,843 bp) revealed the presence of 27 tRNA genes. Proteins encoded by the vB_Sen-IAFB3822 phage were divided into functional modules related to (i) phage structure/assembly, (ii) DNA replication/modification/regulation, (iii) phage lysis, and (iv) DNA packaging into the capsid. No genes associated with antibiotic resistance or integration into the host genome, markers of temperate bacteriophages, were annotated in the Salmonella phage KKP_3822 genome. Based on morphological features and whole-genome sequence analysis, the newly isolated Salmonella phage KKP_3822 shows the greatest similarity to representatives of tailed phages from the *Caudoviricetes* class, *Demerecviridae* family, and *Epseptimavirus* genus. Genome analysis confirmed the virulent nature of the Salmonella phage KKP_3822, making it a potential candidate for food biocontrol.

## 1. Introduction

*Salmonella* is a Gram-negative, facultatively anaerobic, non-spore-forming rod classified within the *Enterobacterales* order, and can cause gastrointestinal infections or systemic salmonellosis [1,2]. According to the European Food Safety Authority (EFSA) and the European Centre for Disease Prevention and Control (ECDC), salmonellosis is the second most frequently reported foodborne gastrointestinal disease in humans, following campylobacteriosis [3]. According to the above report, among the *Salmonella* serovars acquired in the European Union and associated with human infections, the five most common ones predominate: *S*. Enteritidis, *S*. Typhimurium, monophasic *S*. Typhimurium (1,4,[5],12:i:-), *S*. Infantis, and *S*. Derby. This pathogen primarily resides in livestock, particularly poultry, pigs, and cattle, and can lead to food contamination during production [4]. Moreover, *Salmonella* can also be spread by food production workers, as it is often isolated from the food production environment, especially when hygiene and sanitation standards are not maintained [5,6,7].

Irrational management of antibiotic stewardship (AMS) has led to a situation where, since the beginning of the 21st century, we have been witnessing the post-antibiotic era [8,9]. Antibiotics are becoming increasingly ineffective in treating bacterial infections. The acquisition of antibiotic resistance by bacteria is linked to the presence of antibiotic resistance genes (ARGs) in the environment [10]. The rapid spread of ARGs is largely due to the misuse of antibiotics by plant growers and livestock breeders, with many studies indicating that food serves as a significant reservoir of ARGs [10,11]. An example is the widespread use of streptomycin in fruit cultivation in the 1950s to limit infections of apple and pear trees caused by the bacteria of the *Erwinia amylovora* genus. In some countries outside the EU, such as Israel, Canada, Mexico, and New Zealand, this antibiotic is still an approved antibacterial agent in agriculture [11]. Additionally, in the USA, nearly 80% of antibiotics are used in agriculture [12]. Fields fertilized with natural fertilizers, such as manure and slurry, serve as a source of ARG spread and their transfer to both soil bacteria and those inhabiting aquatic environments [13]. At further stages, resistance determinants can be transferred to humans and animals. As a result of these interconnections, genes responsible for antibiotic resistance have been identified in food products. ARGs have been detected in both meat products and minimally processed plant foods, such as fruits and vegetables. Furthermore, genes conferring antibiotic resistance have also spread due to the intensive activities of the dairy industry [11,14]. Foodborne illnesses caused by the consumption of microbiologically contaminated food and drinking water require the implementation of pharmacotherapy. Alarmingly, nearly 90% of antibiotics prescribed to patients are used unjustifiably, leading to the spread of antibiotic-resistant (AMR) strains, including multidrug-resistant (MDR) strains, and ARGs [15]. Currently, bacterial multidrug resistance is a global issue and affects all parts of society (One Health approach). The One Health approach is crucial for addressing AMR because resistant pathogens can rapidly spread across healthcare settings, animals, food sources, and the environment (including soil and water). This widespread transmission complicates the treatment of infections in both humans and animals, heightening the risk of disease transmission, severe illness, and death [16,17]. In order to protect public health and promote the rational use of antibiotics, legal regulations regarding their use have been introduced. Since 2006, the EU has banned the use of antibiotics as growth promoters in livestock production [18,19]. The problem of antibiotic resistance has become the subject of actions taken by the European Parliament and the Council of the European Union. Within the framework of the Community Health Program, antibiotic resistance, alongside tuberculosis, HIV infections, and influenza, is considered one of four priority issues [20]. All EU member states are required to develop national action programs in these priority areas. In Poland, the National Program for Antibiotic Protection has been in place for many years, with some of its tasks being implemented since 2021 under the National Health Program for 2021–2025 [21].

The increasing spread of multidrug-resistant strains and infections caused by MDR bacteria currently poses a serious public health problem. Therefore, there is an urgent need to develop alternative, effective antibacterial agents that could replace antibiotic therapy. In recent years, attention has been drawn to the possibility of using bacteriophages as natural enemies of bacteria. Bacteriophages, as prokaryotic viruses, have the ability to replicate their particles only in bacterial cells, so they do not pose a threat to human, animal, and other eukaryotic cells [22,23]. Bacteriophages are part of the human gut microbiome [24] and are isolated from blood, the urogenital and respiratory tracts, skin, and cerebrospinal fluid [25]. The advantages of using bacteriophages also include their ability to increase their dose automatically; they are self-replicating and accumulate where they have access to their bacterial hosts [26]. In addition, phages are effective against bacterial biofilms, which allows access to other antibacterial agents [27,28]. Bacteriophages are also characterized by high specificity, which may be both their advantage and disadvantage. Unlike broad-spectrum antibiotics, bacteriophages are not a threat to the bacterial microbiota of the gastrointestinal tract (no intestinal dysbiosis effect). Due to their activity directed at a strictly defined species or strain of bacteria, biocontrol in the agro-food industry requires the development of preparations containing a cocktail of different phages with the broadest possible spectrum of activity against bacterial hosts [29]. On the other hand, the strain specificity of bacteriophages limits their destructive activity against bacteria used in the biotechnological or food industry, e.g., starter cultures in the dairy sector [30,31]. The use of cocktails containing many bacteriophages and/or polyvalent phages (i.e., with an increased range of bacterial hosts) may lead to complex pharmacological phenomena and induce an antagonistic effect, resulting in bacteriostatic instead of bactericidal activity [29]. When developing biopreparations for the agro-food industry and bioremediation, only virulent phages should be sought and typed [32]. Due to the nature of phage integration with the bacterial host, phages may participate in horizontal gene transfer (HGT) and ARG transfer in the lysogenic cycle [33,34], which is an undesirable phenomenon.

This study aimed to isolate a bacteriophage targeting indigenous MDR *Salmonella* strains, followed by their biological, morphological, and genomic characterization.

## 2. Results and Discussion

### 2.1. Bacterial Host Strains

All bacterial strains used in this research came from the Culture Collection of Industrial Microorganisms—Microbiological Resource Center (in Polish: Kolekcja Kultur Drobnoustrojów Przemysłowych—Centrum Zasobów Mikrobiologicznych; KKP) of the Department of Microbiology at the Prof. Wacław Dabrowski Institute of Agricultural and Food Biotechnology—State Research Institute (IAFB; Warsaw, Poland). MDR *Salmonella enterica* subsp. *enterica* serovar Manchester (in short: *S*. Manchester) strain KKP 1213 was used to isolate the bacteriophage. Although *S*. Manchester is not one of the most common serovars in the EU, legal regulations imposed on EU member states concern monitoring of the *Salmonella* genus regardless of the serovar. Due to the MDR of this strain associated with the food chain (it was obtained in 2009 from caraway seeds), it was decided to search for bacteriophages specific to this serovar.

Data on the phenotypic and genotypic antibiotic resistance profiles were presented in the previous article [35]. Among the 28 antibiotics tested, *S*. Manchester strain KKP 1213 exhibited a resistance profile for 10 chemotherapeutic agents (i.e., piperacillin, piperacillin/tazobactam, ceftaroline, ceftolozane/tazobactam, ceftriaxone, ertapenem, ofloxacin, amikacin, gentamycin, and tobramycin) belonging to five different antibiotic classes (i.e., penicillins, cephalosporins, carbapenems, fluoroquinolones, and aminoglycosides).

The genome of the primary bacterial host for the phage was sequenced, and the fully assembled genome was deposited in the GenBank database under the CP121296 accession number. The *S*. Manchester strain KKP 1213 genome is 4,578,607 bp in length with a G+C content of 52.2% (Table 1).

Forty ARGs were located and seventy-two putative HGT events were predicted in its genome. Subunits associated with drug efflux pumps were also identified. Efflux pumps contribute to antibiotic resistance by decreasing the concentration of certain antibiotic compounds through their export from the cell across the membranes into the external environment [37]. AcrAB–TolC efflux pump family members, namely, the transporter protein in the inner membrane (AcrB), the auxiliary periplasmatic protein (AcrA), and the activator (MarA), were identified [4]. AcrAB–TolC belongs to the resistance–nodulation–division (RND) efflux pump superfamily [37]. The physiological roles of the AcrAB–TolC efflux pump in *Salmonella* are cell adhesion, cell invasion, virulence, biofilm formation, and cell metabolism [38]. The substrates for the AcrAB–TolC efflux pump are nalidixic acid, ciprofloxacin, doxorubicin, chloramphenicol, quinolones, novobiocin, sulphonamides, macrolides, bile salts, tetracyclines, and aminoglycosides. The second identified family of efflux pump proteins in *S*. Manchester strain KKP 1213 is the ATP-binding cassette (ABC)-type transporter, which is a transport system that plays a role in virulence, host–pathogen interactions, and nutrient absorption [37].

Additionally, a total of 322 regions associated with mobile genetic elements (MGEs) were identified. Among these, 106 were linked to phages, 42 to transfer processes, 33 to integration/excision, 114 to replication/recombination/repair, and 27 to stability/transfer/defense mechanisms. In the *S*. Manchester strain KKP 1213 genome, two CRISPR arrays were predicted; however, no Cas proteins associated with these arrays were identified. The detected *hsdM* and *hsdS* genes indicate the presence of a type I restriction–modification (R–M) system. The clustered regularly interspaced short palindromic repeats (CRISPR)–Cas system provides bacteria with adaptive immunity against invading MGEs. *Salmonella* possesses a type I–E CRISPR–Cas system comprising two CRISPR arrays and one *cas* operon [39]. The CRISPR–Cas system is thought to be involved in the development of antibiotic resistance in bacteria [40].

**Figure 1:**
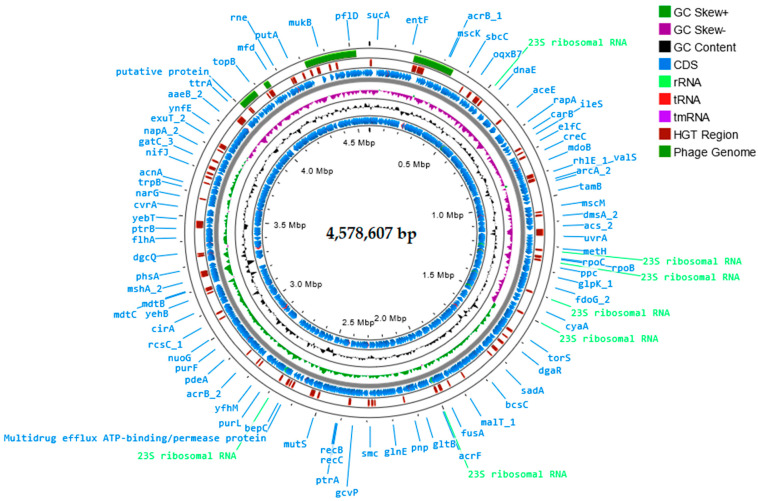
Genome organization map of *S*. Manchester strain KKP 1213 generated using the Proksee program [42].

Furthermore, in the *S*. Manchester strain KKP 1213 genome, four regions (210,309 bp, 77,689 bp, 165,201 bp, and 21,542 bp, respectively) and 160 prophage genes were detected, including three related to integration (Figure 1). Prophages can aid in the transfer of ARGs and virulence factors, impacting the ecological fitness, physiology, diversity, evolution, and pathogenicity of infected *Salmonella* strains. This occurs through HGT involving various interactions between *Salmonella* and prophages, as well as between different prophages, thereby enhancing genetic diversity within the pathogen [41].

In summary, the analysis of the *S*. Manchester strain KKP 1213 genome revealed multidrug resistance in this pathogen, the presence of numerous mechanisms of drug resistance, and phage resistance, which justifies the feasibility of searching for a bacteriophage targeting this serovar.

### 2.2. Lytic Spectrum of the Examined Phage

Salmonella phage KKP_3822 targeting MDR *S*. Manchester strain KKP 1213 was isolated from raw municipal sewage in 2022. The amplified phage lysate (phage titer ~10^7^ PFU mL^–1^) was used to define the bacterial host range via the spot test (Table 2).

Most of the phage lysis zones observed were cloudy plaques. The spot test revealed that the lysate of Salmonella phage KKP_3822 exhibited a broad range of activity, inhibiting growth in 90.7% (49 out of 54) of the tested *Salmonella* strains. Additionally, it demonstrated effectiveness against six saprophytic strains, including three from *Escherichia coli* and three from *Enterobacter cloacae* species. However, pathogenic bacteria outside of *Salmonella*, including both Gram-negative (*Pseudomonas aeruginosa*) and Gram-positive (*Listeria monocytogenes*, *Staphylococcus aureus*) species, showed resistance to the tested phage.

Assessing the host range is a crucial step in characterizing bacteriophages, enabling the selection of bacterial viruses for the creation of broad-spectrum biopreparations [43]. Phage cocktails help prevent the development of bacterial resistance to phage infections [44]. By incorporating bacteriophages with overlapping host ranges into a biopreparation, greater biodiversity is achieved, enhancing efficacy, particularly when bacteria develop resistance to one of the phages. A combination of different phages with a broad host range can serve as an alternative to broad-spectrum antibiotics [45]. Although bacteriophages are generally highly specific, some can infect bacteria across an entire genus or even family. For instance, phage lysates from other salmophages (i.e., Salmonella phage KKP_3829 and Salmonella phage KKP_3830) isolated by Wójcicki and coworkers (2023) demonstrated, similar to Salmonella phage KKP_3822, activity against saprophytic strains of *E. coli* and *E. cloacae* species [46]. Understanding the intricate dynamics of bacteria–phage interactions is essential for developing effective biocontrol strategies and phage therapies [47]. The activity of our salmophage against saprophytic bacteria is a beneficial trait, potentially allowing for the replication of phage particles in non-pathogenic hosts. Future studies should include the efficiency of plating (EOP) test to confirm the activity of phage particles rather than just the phage enzymes present in the lysate.

### 2.3. Phage Physiological Parameters

**Figure 2:**
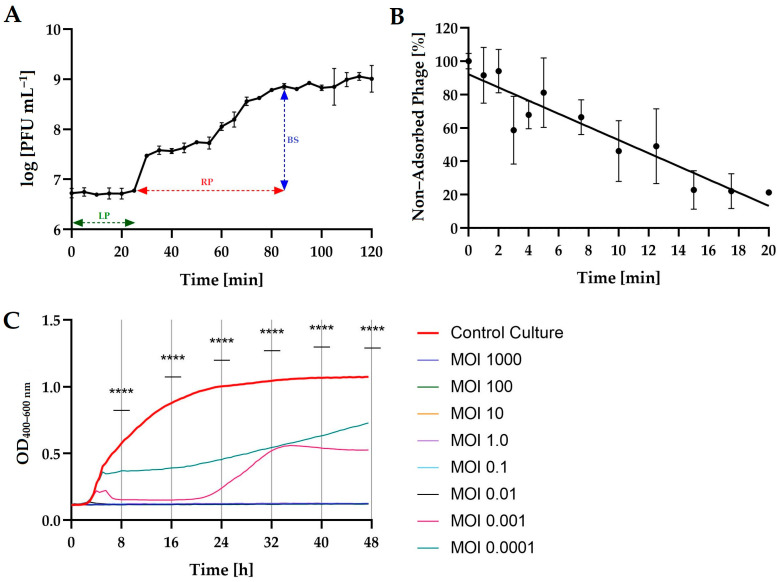
(**A**) One-step growth curve of Salmonella phage KKP_3822 at MOI = 0.1. Data points represent the average, and error bars indicate the mean with standard deviation (±SD) of the mean phage titers from three repetitions (*n* = 3). The chart symbols are as follows: LP—latent period; RP—rise period (burst time); BS—burst size. (**B**) Kinetic adsorption of Salmonella phage KKP_3822 at MOI = 0.1. Data points represent the average, with error bars showing the mean and standard deviation (±SD) of the mean phage titers from three repetitions (*n* = 3). (**C**) Growth curves of the bacterial host strains (*n* = 10) treated with Salmonella phage KKP_3822 at various MOI ratios compared to the control culture (red bold line). **** means a significant difference (*p* ≤ 0.0001) of the control culture (with Salmonella) vs. the phage-treated cultures.

For Salmonella phage KKP_3822, a one-step growth curve (Figure 2A), the kinetic of adsorption (Figure 2B), and host bacterial growth curves (Figure 2C) at different phage multiplicity of infection (MOI) ratios were determined.

To assess the growth characteristics of Salmonella phage KKP_3822, we conducted a one-step growth curve analysis (Figure 2A). The latent period of this salmophage was found to be 25 min, with a rise period (burst time) of 60 min and a burst size of 21 ± 1 PFU cell^−1^. Both the latent period and burst size are critical factors in evaluating a phage’s potential suitability for food biocontrol [48]. Research indicates that a large burst size and a short latent period are positively associated with efficient bacterial inactivation [49]. Other studies on salmophages have reported similar latent periods but different burst sizes. In the case of Salmonella phage BIS20, the latency period was 20 min with a burst size of 110 ± 7 PFU cell^−1^ [50]. In a study by the team of Mondal and coworkers (2022), Salmonella phage STWB21 was analyzed in a one-step growth assay on various *Salmonella* serovars (*S*. Typhi, *S*. Paratyphi, *S*. Typhimurium, and *S*. Enteritidis). The latency periods were shown to be 25, 55, 50, and 35 min, respectively, while the burst sizes were 101, 53, 163, and 224 PFU cell^−1^, respectively [51].

After 5 min of incubation, the average adsorption level was 18.8 ± 14.8%. By the end of the experiment (20 min), Salmonella phage KKP_3822 significantly increased the number of adsorbed particles to 78.6 ± 0.7% (Figure 2B). The calculated adsorption rate constant (*k*) was 2.23 × 10^−9^ mL min^−1^. The adsorption rate is an another critical factor to consider when selecting phages for food biocontrol. In the study conducted by Kwon and coworkers (2021) on Salmonella Jumbo-Phage pSal-SNUABM-04, the adsorption test showed that 95% of the phage was adsorbed within 15 min, while the phage adsorption constant *k* was 3.76 × 10^−10^ mL min^−1^. Compared to our results, Salmonella Jumbo-Phage pSal-SNUABM-04 showed better adsorption in a shorter time [52].

The activity of Salmonella phage KKP_3822 against its primary host was assessed. Growth curves for the bacterial host strain were established by measuring the optical density in relation to the bacterial titer (unpublished data). From these standards, various ratios of bacteria to phages (MOIs) were determined. The results indicated that Salmonella phage KKP_3822 inhibited the growth of the bacterial host to varying degrees depending on the MOI (Figure 2C). Complete inhibition of the *S*. Manchester strain KKP 1213 was observed at a miMOI of 0.01. The minimum inhibitory MOI (miMOI) is defined as the lowest concentration of bacteriophages needed for 100% bacterial inhibition, while the MOI represents the ratio of bacteriophages to primary host bacteria [53]. By analyzing the miMOI for a given bacteriophage, the most effective MOI (the best MOI) for application can be identified. Research data indicates that variations in miMOI among bacteriophages are related to their adsorption rates [54]. In the case of phage vB_SalP_TR2 against *S*. Albany, bacterial culture growth inhibition was observed, but no obvious differences in bacterial inhibition were observed between different MOI ratios. The best inhibitory effects were observed at MOI = 0.01 and 0.001. In the case of higher infection rates (i.e., MOI = 100, 10, 1, 0.1), inhibition was followed by a dominant increase in optical density [55]. MOI-dependent growth restriction and bacterial regrowth were also observed in Salmonella phage ph 2–2 following infection with *Salmonella* Paratyphi and *Salmonella* Typhimurium strains [56].

### 2.4. Impact of Selected Factors on the Maintenance of Salmonella Phage KKP_3822 Activity

**Figure 3:**
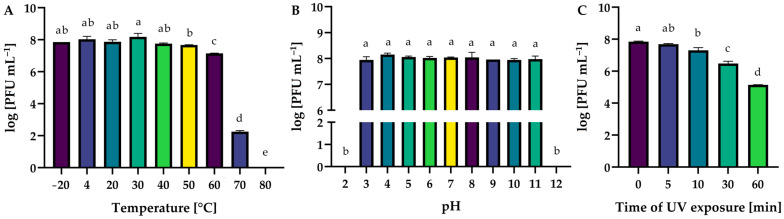
Influence of: (**A**) temperature, (**B**) active acidity (pH), and (**C**) UV exposure time on Salmonella phage KKP_3822 activity. Letters a, b, c, d, and e indicate homogenous groups at a significance level of *p* ≤ 0.05. Sample measurements were performed in triplicate (*n* = 3).

The activity of Salmonella phage KKP_3822 upon exposure to a wide range of temperatures (from −20 °C to 80 °C), active acidities (pH from 2 to 12), and UV exposure times (0, 5, 10, 30, and 60 min) was evaluated (Figure 3).

Temperature and active acidity (pH) are critical factors affecting bacteriophage viability and activity in the environment [57]. They significantly influence the phages’ ability to attach to, penetrate, and replicate within bacterial host cells [58,59]. Salmonella phage KKP_3822 was stable up to 40 °C. The temperature of 70 °C inactivated 99.999% of the virion particles (*p* ≤ 0.05), while at 80 °C the phages were inactive (Figure 3A). Salmophages remained stable across a broad pH range, from 3 to 11. Phage virions were completely inactivated by extreme pH values of 2 and 12 (*p* ≤ 0.05; Figure 3B). The exposure of Salmonella phage KKP_3822 to UV radiation significantly decreased its activity in proportion to the exposure time (*p* ≤ 0.05; Figure 3C). It should be noted that an hour of UV radiation exposure is not a reliable method for fully inactivating phage virion particles. Evaluating phage resistance to environmental factors is crucial for understanding their potential synergistic effects when used as biological agents alongside other physical (e.g., temperature, UV) or chemical (e.g., organic acid solutions) methods in food preservation or the disinfection of production lines [60]. The stability of a bacteriophage under changing environmental conditions is an individual trait, depending on its structure and genome [61]. In a study conducted by Shang and coworkers (2021) on the Salmonella phage vB_SalP_TR2, the phage was highly active during an hour-long incubation in the temperature range from 2 °C to 60 °C and the pH range from 4 to 11 [55]. Similar results were obtained for Salmonella phage L223, where high activity was demonstrated in the temperature range from 20 °C to 60 °C and the pH range from 4 to 11 [62]. In other studies on Salmonella phage KKP_3829 and Salmonella phage KKP_3830, a slight decrease in the titer (reduction of about 2.5 log) of the phage was also observed after irradiation for 1 h [46]. The results obtained by Zhao and coworkers (2022) on 30-min UV exposure showed that Salmonella phage ph 2–2 still showed high antibacterial activity and high titers [56].

### 2.5. Determination of Morphological Features of Salmonella Phage KKP_3822 and Its Plaques

**Figure 4:**
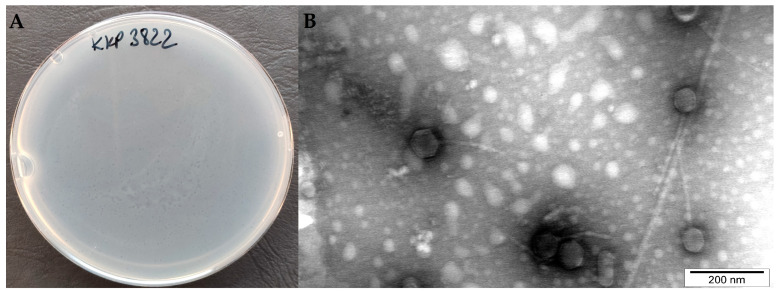
(**A**) Plaque morphology observed on agar plates; (**B**) electron micrograph from the transmission electron microscopy showing the morphology of Salmonella phage KKP_3822 (120,000×).

Salmonella phage KKP_3822 produced small transparent plaques measuring less than 1 mm in diameter, with no visible ‘halo’ zone (Figure 4A). The absence of a halo is thought to indicate a lack of phage-encoded bacterial exopolysaccharide (EPS)-degrading depolymerase, which is crucial for damaging host bacterial cell membranes and disrupting biofilm structures [63,64]. Transmission electron microscopy (TEM) provided insight into the morphology of the virions (Figure 4B), revealing phage particles with a siphovirus morphology. Salmonella phage KKP_3822 has a complex structure and is classified as a tailed phage, with total dimension of 188.9 nm, containing an isometric head (96.7 nm length by 45.6 nm width) and a long non-contractile tail (92.2 nm length by 14.2 nm width). Generally, large phages diffuse slowly in the agar medium, resulting in smaller plaques [65], a trend confirmed for Salmonella phage KKP_3822.

### 2.6. Genome Sequencing and Bioinformatics Analysis of Salmonella Phage KKP_3822

Newly isolated Salmonella phage KKP_3822 was deposited in the KKP of the Department of Microbiology at the IAFB. The entire genome of Salmonella phage KKP_3822 has been sequenced, submitted, and deposited in the GenBank database with the accession number OQ674104.

**Figure 5:**
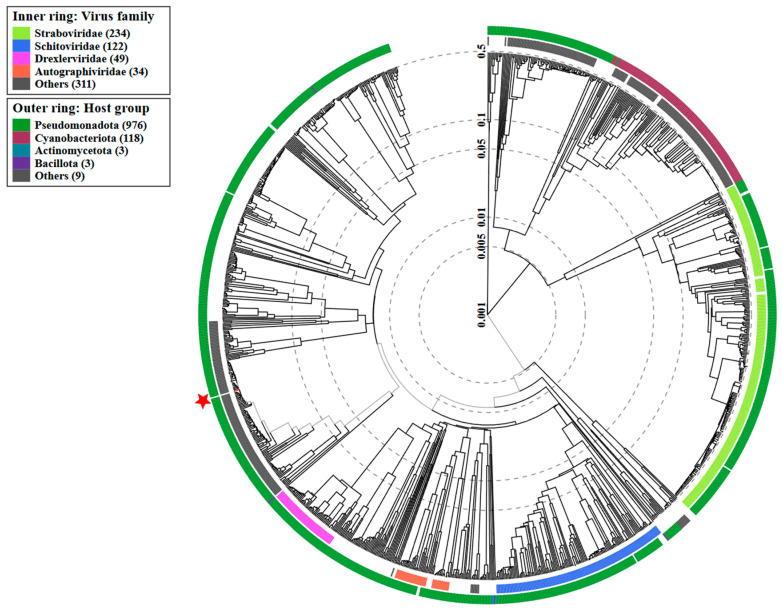
The viral proteomic tree of Salmonella phage KKP_3822 and other phage genomes is shown in a circular layout. The branch representing the studied phages is marked with an asterisk. Color-coded rings indicate virus families (inner rings) and host groups (at the phylum level; outer rings). This tree was constructed using BIONJ based on genomic distance matrices and midpoint rooting. Branch lengths are log-scaled. Sequence and taxonomic data were sourced from Virus-Host DB [68], and the tree was generated using the ViPTree server [69].

Genomic characterization and taxonomic classification of the newly isolated Salmonella phage KKP_3822 were performed according to the latest International Committee on Taxonomy of Viruses (ICTV) guidelines from January 2023 [66]. According to the Bacterial Viruses Subcommittee (BVS) of the ICTV, two phages are classified under the same species if their genomes share more than 95% identity, while a genus is defined as a cohesive group of viruses that exhibit more than 70% nucleotide identity across the entire genome [67]. Genome analysis confirmed the results obtained by TEM, which suggested that Salmonella phage KKP_3822 belongs to the *Caudoviricetes* class. Figure 5 presents a proteomic tree created using the BIONJ program, based on TBLASTX genomic sequence comparisons with other phage genomes available in the Virus-Host DB [68].

**Figure 6:**
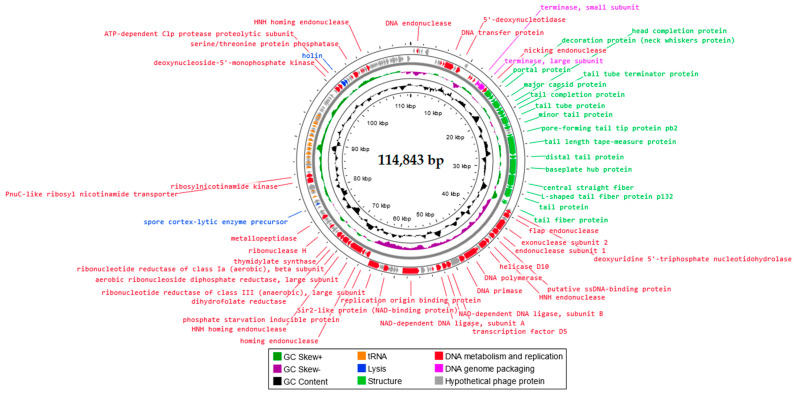
Genome organization map of Salmonella phage KKP_3822 generated in the Proksee program [42].

Genome sequencing of Salmonella phage KKP_3822 revealed that it has linear double-stranded DNA (dsDNA). A map illustrating the genome organization of Salmonella phage KKP_3822, which belongs to the *Demerecviridae* family and *Epseptimavirus* genus, is presented in Figure 6. Salmonella phage KKP_3822 has a genome length of 114,843 bp and a total G+C content of 39.8%. The identified functional proteins have been categorized into four groups based on their functions: those related to lysis, structure, DNA metabolism and replication, and DNA genome packaging. Of the 170 predicted open reading frames (ORFs), 69 are associated with genes encoding proteins of known function, while 101 ORFs encode hypothetical proteins with unknown functions. Moreover, in the Salmonella phage KKP_3822 genome, 27 tRNA regions were located. The presence of tRNAs in phage genomes has been observed to favor the replication cycle, possibly providing an advantage in expanding their host range and allowing large phages to replicate their particles [70]. tRNA found in phage genomes aligns with codons that are frequently utilized by the phage but less so by the bacterial host during infection. This alignment may boost the expression of late phage genes that encode structural proteins, including capsid and tail proteins [71]. Some phages, particularly those with compact genomes, do not possess a tRNA coding sequence. This suggests that they rely on the host bacteria’s genetic expression system for replication and translation, utilizing bacterial tRNA to facilitate these processes within host cells [72].

In the Salmonella phage KKP_3822 genome, proteins associated with lysis, i.e., holin and spore cortex-lytic enzyme precursor, were predicted. Holins are small (>100 nm in diameter) hydrophobic hole-forming proteins [73]. Holins are located in the inner membrane of the bacterial cell and, by creating holes in the cell membrane, allow endolysins (another group of phage lytic enzymes) to enter the bacterial periplasm [74]. In the Salmonella phage KKP_3822 genome, no ARGs, genes encoding virulence factors, integrase, recombinase, or repressors, which are markers of lysogenic (temperate) bacteriophages, were identified. This allows us to assume that Salmonella phage KKP_3822 is a strictly lytic (virulent) virus.

**Figure 7:**
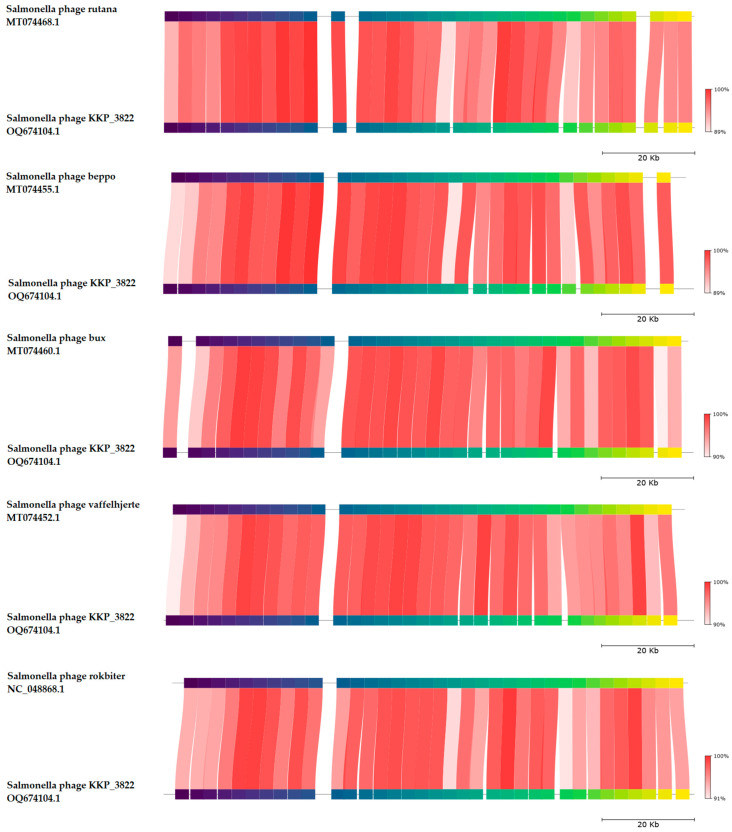
A comparison of the Salmonella phage KKP_3822 genome sequence with five related phage genomes reveals co-linearity, as identified through TBLASTX using FastANI v1.3.3 software [75] and Proksee program [42]. Homologous regions found in the TBLASTX search are linked by segments colored according to orthologous matches from fragments of the query sequence. The color bar indicates the percentage of TBLASTX identity.

BLASTn similarity searches conducted for Salmonella phage KKP_3822 and phages available in GenBank showed a 98.53% nucleotide similarity with 90% query coverage to Salmonella phage rutana (GenBank Acc. No. MT074468.1), 98.30% nucleotide similarity with 88% query coverage to Salmonella phage beppo (GenBank Acc. No. MT074455.1), 97.49% nucleotide similarity with 87% query coverage to Salmonella phage bux (GenBank Acc. No. MT074460.1), 97.30% nucleotide similarity with 88% query coverage to Salmonella phage vaffelhjerte (GenBank Acc. No. MT074452.1), and 97.22% nucleotide similarity with 90% query coverage to Salmonella phage rokbiter (GenBank Acc. No. NC_048868.1) (Figure 7 and Table 3).

**Figure 8:**
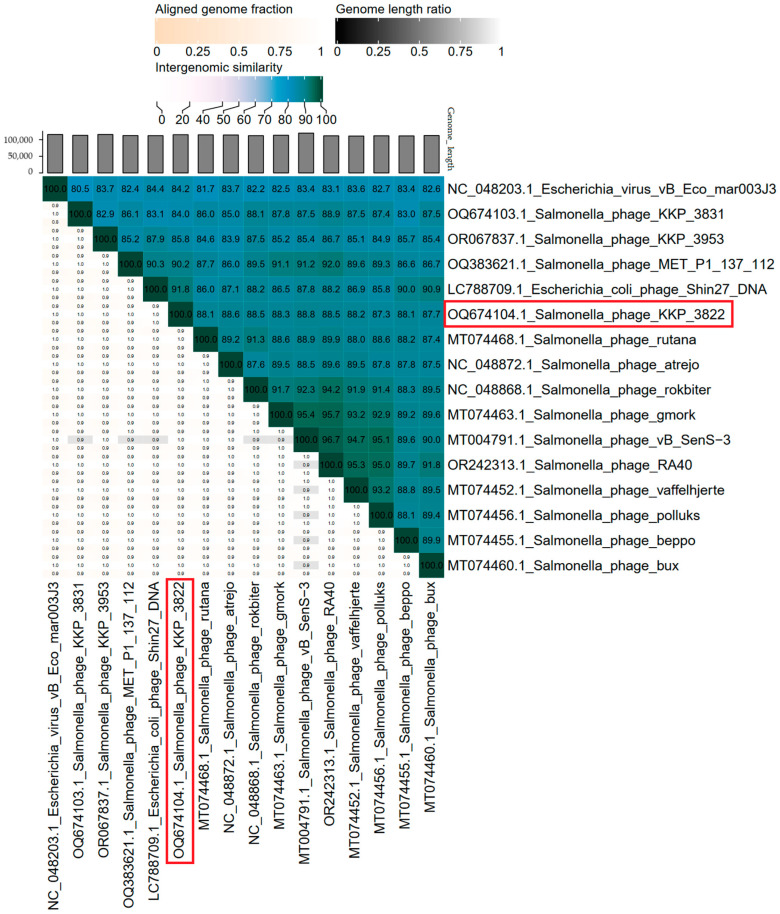
The whole-genome comparison and clustering of Salmonella phage KKP_3822 (highlighted in a red frame) and its 15 closest relatives were conducted using VIRIDIC v1.1 [76]. The right half of the heatmap uses various shades of blue-green to represent the intergenomic similarities (%) between each pair of genomes, as indicated above the heatmap with corresponding numerical values. The left half of the heatmap displays three key metrics for each genome pair: the aligned fraction of the genome in the row (top value), the genome length ratio between the two genomes (middle value), and the aligned fraction of the genome in the column (bottom value).

The overall nucleotide sequence identity between Salmonella phage KKP_3822 and its 15 closest relatives was calculated using VIRIDIC v1.1 (Figure 8). Our phage showed the highest intergenomic similarity (91.8%) to Escherichia coli phage Shin27 DNA (GenBank Acc. No. LC788709.1).

## 3. Materials and Methods

### 3.1. Bacterial Host Strains

In this article, an attempt was made to isolate a phage specific for MDR *Salmonella enterica* subsp. *enterica* serovar Manchester strain KKP 1213 (in short: *S*. Manchester strain KKP 1213). The bacterial genome was sequenced in genXone SA (Złotniki, Poland). For this purpose, bacterial genetic material was isolated using the Genomic Mini AX Bacteria kit (A&A Biotechnology, Gdynia, Poland) according to the manufacturer’s protocol. According to the manufacturer’s protocol, the DNA library was prepared using Rapid Barcoding Kit v10 reagents (Oxford Nanopore Technologies, Oxford, UK). A sequencing depth of at least 50× genome coverage was assumed. Next-generation sequencing (NGS) was performed using nanopore technology on the GridION X5 sequencing device (Oxford Nanopore Technologies, Oxford, UK) on flow cell R9.4.1 under the control of MinKnow v22.10.5. Bases were called with neural network-based tool Guppy v6.3.8 Basecaller (super-accurate basecalling; Oxford Nanopore Technologies, Oxford, UK), followed by barcode demultiplexing, also using Guppy Barcoder v6.3.8 (Oxford Nanopore Technologies, Oxford, UK), generating a fastq file. De novo assembly of the genome was performed in Flye v2.8.1 software [77], while annotation of the bacterial genome was performed in Prokka 1.14.6 v1.1.1 software [78], Bakta v1.8.2 software [36], and the Reference Sequence (RefSeq) v6.7 database at the National Center for Biotechnology Information (NCBI) [79]. Proksee program [42] was used to visualize the bacterial genome. RGI 6.0.3 v1.2.1 software was used to search for ARGs in the bacterial genome [80]. Alien_hunter 1.7 v1.1.0 software predicted putative HGT events [81]. MGEs were searched in mobileOG-db (beatrix-1.6) v1.1.3 software [82]. CRISPR arrays and their associated Cas proteins were analyzed in CRISPRCas-Finder 4.2.20 v1.1.0 software [83]. VirSorter 2.2.4 v1.1.1 [84] and Phigaro 2.3.0 v1.0.1 [85] software were used to detect and characterize prophage regions in the bacterial genome. The *S*. Manchester strain KKP 1213 genome was deposited in the GenBank database.

### 3.2. Isolation, Purification, and Bacteriophage Propagation

Phage isolation was performed using raw municipal sewage from the Czajka Wastewater Treatment Plant (Warsaw, Poland). The procedure described by Wójcicki and coworkers [46] was used. A total of 25 mL of municipal sewage was centrifuged at 10,000× *g* (20 °C for 10 min) to separate organic and mineral particles from bacteria and potential bacteriophages. The supernatant was then filtered through a 0.22 μm syringe filter (Sartorius, Göttingen, Germany). Next, 20 mL of the filtered supernatant containing bacteriophages was transferred into 20 mL of double-concentrated Luria–Bertani broth (BTL, Lodz, Poland). To this, 1 mL of an overnight bacterial culture in Luria–Bertani broth was added, and the mixture was incubated at 37 °C for 24 h. Following incubation, the culture was centrifuged at 8000× *g* for 10 min to separate the bacteria from the proliferated bacteriophages. The resulting supernatant was filtered through a 0.45 μm syringe filter (Sartorius, Göttingen, Germany).

Phage concentration in the lysate was determined by the double-layer agar plate method [86] with our modifications by determining the phage titer. Briefly, a series of tenfold dilutions of phage lysates were prepared using a multi-well plate (well capacity: 2 mL). To 500 µL of each phage lysate dilution, 100 µL of the host bacterial strain was then added and left for 20 min. The entire phage with bacteria suspension was transferred to a Petri dish overlaid with a layer of nutrient agar and covered with 4 mL of soft agar. The solidified dishes were incubated at 37 °C for 24 h.

Single-phage plaque was cut with a scalpel and purified in saline magnesium (SM) buffer according to the Mirzaei and Nilsson [87] method. The purification procedure was performed in four rounds of single-plaque passage to ensure that the isolate represented a clonal phage population. Furthermore, after being filtered through a 0.45 μm syringe filter, each lysate was inoculated to assess potential contamination with bacterial cells.

### 3.3. Bacterial Host Range for the Tested Phage

To assess the lytic spectrum of the phage, 81 bacterial strains were used (Table 2), consisting of a group of 54 *Salmonella enterica* strains and a group of 27 strains other than *Salmonella* spp. Bacterial strains were isolated and deposited in the Culture Collection of Industrial Microorganisms—Microbiological Resource Center of the Department of Microbiology at the Prof. Wacław Dąbrowski Institute of Agricultural and Food Biotechnology—State Research Institute (IAFB; Warsaw, Poland). Taxonomic identification of most of the bacteria used was established during previous studies described in our articles [35,46,88]. The ability of the phage (phage titer ~10^7^ PFU mL^−1^) to infect bacteria with different strains was determined using a spot test [49]. Briefly, 100 µL of bacterial strain was poured onto Petri dishes with nutrient agar and covered with 4 mL of soft agar. After solidification, 5 µL of phage lysate was added to the agar surface. The range of lytic activity of all bacterial strains was determined at 37 °C. After 24 h of incubation, transparent spots/plaques on bacterial lawns were recorded as corresponding to phage sensitivity according to the following signs: “++”—transparent plaques; “+”—turbid plaques; “−”—no plaques (non-susceptible bacterial strain). All experiments were performed in triplicate.

### 3.4. One-Step Growth

A one-step growth curve experiment was performed to evaluate the latent period, rise period (burst time), and burst size following the method described by Islam and coworkers [49] and Shakeri and coworkers [89], with Wójcicki and coworkers’ [88] modifications. Experiments were performed in triplicate using the double-layer agar plate method. The latent period was defined as the time elapsed between absorption and the onset of the first burst. Burst size was quantified as the ratio of the final number of phage particles to the initial number of bacterial hosts at the start of the experiment [90,91].

### 3.5. Phage Adsorption Time

To evaluate the phage adsorption constant and investigate the kinetic of phage adsorption to host bacterial cells, we followed the procedure outlined by Cieślik and coworkers [92]. The quantity of free, non-adsorbed phage particles in the supernatant was quantified using the double-layer agar plate method, conducted in triplicate. The initial phage count immediately after mixing with the host bacterial suspension was taken as 100% of the free phage particles, serving as a reference point for comparison with subsequent titers. Additionally, the adsorption rate constant (*k*) was calculated.

### 3.6. Changes in the Growth Kinetics of Bacterial Hosts After Phage Infection

Growth kinetics of primary bacterial cultures following phage infection compared to control cultures were measured using the Bioscreen C Pro automated growth analyzer (Yo AB Ltd., Growth Curves, Helsinki, Finland) according to the procedure described previously [46,54]. MOI values of 1000, 100, 10, 1.0, 0.1, 0.01, 0.001, and 0.0001 were used in this study. The minimum inhibitory multiplicity of infection (miMOI) of bacteriophages was determined. All experiments were performed with ten replicates.

### 3.7. Influence of Selected Factors on the Preservation of the Phage Activity

The phage activity was assessed under various conditions including temperature, pH values (active acidity), and UV exposure time. To evaluate the phage lysates’ effectiveness at different temperatures (i.e., −20 °C, 4 °C, 20 °C, 30 °C, 40 °C, 50 °C, 60 °C, 70 °C, and 80 °C), 100 µL of the phage suspension was added to 9.9 mL of physiological saline with a pH of 7.0. The mixture was then incubated for 1 h at the designated temperatures [93]. For testing the phage lysates’ activity across a wide range of pH values, 100 µL of the lysate was added to test tubes containing 9.9 mL of sterile physiological saline (0.85% NaCl) adjusted to specific pH values between 2 and 12. Mixtures were incubated at 20 °C for 1 h. Additionally, the impact of UV radiation was investigated by exposing phage lysate to UV light for varying durations of 0, 5, 10, 30, and 60 min. All experiments were conducted with three independent replicates, and the phage concentration, expressed as the phage titer, was determined using the double-layer agar plate method.

### 3.8. Electron Micrographs of Phage Particles

Transmission electron microscopy (TEM) was used to determine the morphological features of the bacteriophage. Samples were prepared according to the procedure described previously [46]. Slides were negatively stained with uranyl acetate and visualized under a JEOL JEM–1220 transmission electron microscope (Japan Electron Optics Laboratory Co., Ltd., Tokyo, Japan) at a magnification of 60,000–150,000× and a voltage of 80 kV [91]. Images were sized using SightX v1.2.3.537 software (Japan Electron Optics Laboratory Co., Ltd., Tokyo, Japan).

### 3.9. Phage Genome Sequencing and Bioinformatics Analysis

Phage genomic DNA was isolated using the PureLink™ Viral RNA/DNA Mini Kit (Thermo Fisher Scientific Inc., Carlsbad, CA, USA) according to the manufacturer’s protocol, with modifications described previously by Wójcicki and coworkers [46].

The isolated genomic phage DNA was forwarded to genXone SA (Złotniki, Poland) for NGS in the WGS (whole genome sequencing) application. DNA libraries were prepared according to the manufacturer’s protocol using Rapid Barcoding Kit reagents (Oxford Nanopore Technologies, Oxford, UK). A sequencing depth of at least 50× genome coverage was targeted. NGS sequencing was conducted via nanopore technology on the GridION X5 sequencer (Oxford Nanopore Technologies, Oxford, UK) under the control of MinKnow v22.10.5. Bases were called with Guppy v6.3.8 Basecaller (Oxford Nanopore Technologies, Oxford, UK), followed by barcode demultiplexing, also using Guppy Barcoder v6.3.8, generating a fastq file for each barcode. De novo phage genome assembly was carried out using Flye v2.8.1 software [77], and the genome was annotated using Phanotate v1.5.0 [94] and PhaGAA server (http://phage.xialab.info/home, accessed on 10 May 2024) [95]. The visualization of the phage genome was achieved through Proksee program [42]. The viral proteomic tree of the phage genome was calculated using BIONJ based on genomic distance matrixes and mid-point rooting and was represented in the circular view. Branch lengths were log-scaled. The sequence and taxonomic data were based on Virus–Host DB [68]. The tree was generated using the ViPTree v4.0 server [69]. Comparative genomic analysis of the newly isolated phage against five related phage genomes exhibiting co-linearity was performed using TBLASTX using FastANI v1.3.3 software [75] and Proksee program [42]. The nucleotide sequence identity of the isolated phage with 15 other phages available in the GenBank database was computed using VIRIDIC v1.1 (intergenomic distance calculator) [76]. The phage genome was subsequently deposited in the GenBank database.

### 3.10. Statistical Analysis

All the experiments were repeated at least three times. All data presented graphically or in tables were subjected to statistical analyses performed using Graph Prism v9.4.1 (GraphPad Software Inc., San Diego, CA, USA) unless otherwise stated.

## 4. Conclusions

As antibiotic resistance becomes an increasing global concern, researchers and the food industry are increasingly interested in using bacteriophages for targeted biocontrol in food safety. In some regions of the world (e.g., USA, Brazil, Switzerland, and Canada) phage biocontrol is widely used in the food industry, while in EU Member States there is still no clear legal regulation in this area. In 2016, the EFSA Panel of Biological Hazards issued a positive opinion on a phage biopreparation (Listex™ P100 by Micreos Food Safety, Wageningen, The Netherlands) targeting *L. monocytogenes* in three categories of ready-to-eat products, namely, meat and poultry, fish and shellfish, and dairy products, but based on scientific research studies, the Expert Panel recommended further studies on the efficacy of the preparation and its dose and additional experiments to investigate the currently unknown mechanisms by which *L. monocytogenes* strains showing resistance to some therapeutic antimicrobials become sensitive to these antimicrobials after developing resistance to P100 bacteriophages [96]. Work on phage therapy (or biocontrol) at the EU level is ongoing [97]. In October 2023, the European Medicines Agency (EMA) published the “Guideline on quality, safety and efficacy of veterinary medicinal products specifically designed for phage therapy” [98]. Furthermore, in August 2024, EFSA published an article entitled “EFSA statement on the requirements for whole genome sequence analysis of microorganisms intentionally used in the food chain”. The microorganisms covered by the document include bacteria, yeasts, filamentous fungi, and viruses (including bacteriophages). For bacteriophages, it is recommended that WGS data be generated for the phage itself and the bacterial host strain in which it is replicated and used for full characterization [99].

Each region of the world has its own unique microbiota, shaped by its environment. This is particularly true for *Salmonella*, which includes various serovars; some are unique to certain countries, while others have never been detected there. In response to this problem, our team focused on isolating bacteriophages that specifically target local multidrug-resistant *Salmonella* strains, thoroughly characterizing these phages in terms of their morphology, biology, and genomic features. Our results suggest that the newly isolated Salmonella phage KKP_3822, comprehensively characterized in this study, holds significant promise as a biocontrol agent for managing *Salmonella* in food settings.

## Figures and Tables

**Table 1:** The *S*. Manchester strain KKP 1213 genome sequence details were obtained with Bakta v1.8.2 software [36].

Genome (GenBank Accession Number)	Genome Length [bp]	G+C Content [%]	CDS	tRNA	tmRNA	rRNA	ncRNA	ncRNA Region	Repeat Region	sORF	gap	oriC	oriV	oriT
*S.* Manchester strain KKP 1213 chromosome (CP121296)	4,578,607	52.2	4271	89	1	22	131	60	2	10	0	2	0	0

**Table 2:** The host range for newly isolated Salmonella phage KKP_3822.

Bacterial Host Strain	GenBank Accession Number	Spot Test	Bacterial Host Strain	GenBank Accession Number	Spot Test
*Salmonella* Strains (*n* = 54)					
*S*. Berta strain KKP 996	ON627842	+	*S.* Typhimurium strain KKP 3079	MW033548	+
*S. enterica* R strain KKP 997	MW046052	+	*S.* Typhimurium strain KKP 3080	MW033536	+
*S*. I (6,8:1,v:-) strain KKP 998	ON764274	+	*S.* Typhimurium strain KKP 3081	MW033602	+
*S*. Oranienburg strain KKP 999	ON627845	+	*S.* Enteritidis strain KKP 3814	ON732733	+
*S*. I (4,12:i:-) strain KKP 1000	ON312999	+	*S.* Enteritidis strain KKP 3815	ON732742	+
*S*. Kunduchi strain KKP 1001	MW332255	+	*S.* Enteritidis strain KKP 3816	ON756119	+
*S*. Muenster strain KKP 1002	ON340716	+	*S.* Enteritidis strain KKP 3817	ON756120	+
*S*. Hadar strain KKP 1003	ON756138	+	*S.* Enteritidis strain KKP 3818	ON756135	+
*S. enterica* R strain KKP 1004	ON627844	+	*S.* Typhimurium strain KKP 3819	ON732745	+
*S*. Senftenberg strain KKP 1005	ON627847	+	*S*. I (4,12:i:-) strain KKP 3820	ON732744	+
*S*. Derby strain KKP 1006	ON764251	−	*S*. Abortusequi strain KKP 3821	ON732827	+
*S*. Mbandaka strain KKP 1007	ON627846	+	*S.* Sandiego strain KKP 3882	OP745459	+
*S*. I (6,8:1,v:-) strain KKP 1008	ON340717	+	Other Non-Pathogenic *Enterobacterales* Strains (*n* = 21)		
*S*. Amsterdam strain KKP 1009	ON764277	+	*Citrobacter freundii* strain KKP 3655	MZ827001	−
*S*. Potsdam strain KKP 1010	ON764279	+	*Enterobacter cloacae* strain KKP 3082	MZ827006	+
*S*. Infantis strain KKP 1039	ON764252	+	*Enterobacter cloacae* strain KKP 3656	OM304355	−
*S. enterica* R strain KKP 1040	ON764280	−	*Enterobacter cloacae* strain KKP 3684	OM281790	+
*S*. Agona strain KKP 1041	ON764253	+	*Enterobacter cloacae* strain KKP 3686	OM281778	+
*S*. Infantis strain KKP 1042	ON798424	+	*Enterobacter ludwigii* strain KKP 3083	MZ827002	−
*S*. Kentucky strain KKP 1043	ON764281	+	*Pantoea agglomerans* strain KKP 3651	OP978292	−
*S*. Muenchen strain KKP 1044	ON764287	+	*Raoultella terrigena* strain KKP 3689	OK085529	−
*S*. Livingstone strain KKP 1045	ON764254	−	*Serratia fonticola* strain KKP 3084	MZ827668	−
*S. enterica* R strain KKP 1113	ON775567	−	*Serratia fonticola* strain KKP 3685	OM281802	−
*S*. Mbandaka strain KKP 1169	ON764259	−	*Serratia fonticola* strain KKP 3692	OM281803	−
*S*. Abony strain KKP 1193	ON764258	+	*Serratia liquefaciens* strain KKP 3654	OP978313	−
*S*. Manchester strain KKP 1213	ON764805	++ ^H^	*Serratia marcescens* strain KKP 3687	OK103977	−
*S*. Manchester strain KKP 1217	ON764807	+	*Escherichia coli* strain KKP 3688	OM281784	+
*S*. Manchester strain KKP 1514	ON756136	+	*Escherichia coli* strain KKP 3691	OM281773	−
*S*. Senftenberg strain KKP 1597	ON461374	+	*Escherichia coli* strain KKP 3707	OM281777	+
*S.* Newport strain KKP 1608	ON312943	+	*Escherichia coli* strain KKP 3800	OM250392	−
*S*. I (6,8:1,v:-) strain KKP 1610	ON313000	+	*Escherichia coli* strain KKP 3801	OM250391	+
*S.* Chandans strain KKP 1611	ON764857	+	*Escherichia coli* strain KKP 3802	OM250393	−
*S*. Manchester strain KKP 1612	ON764858	+	*Escherichia coli* strain KKP 3824	ON303636	−
*S*. Cannstatt strain KKP 1613	ON766359	+	*Escherichia coli* strain KKP 3825	ON303626	−
*S*. Newport strain KKP 1614	ON312941	+	Other Pathogenic Gram-Negative Strains (*n* = 2)		
*S*. Typhimurium strain KKP 1636	ON773156	+	*Pseudomonas aeruginosa* strain KKP 994	OQ302514	–
*S*. Infantis strain KKP 1761	ON798425	+	*Pseudomonas aeruginosa* strain KKP 1593	OK189606	–
*S*. I (6,8:1,-:1,7) strain KKP 1762	ON340720	++	Other Pathogenic Gram-Positive Strains (*n* = 4)		
*S*. Senftenberg strain KKP 1763	ON773159	+	*Listeria monocytogenes* strain KKP 1845	OK663000	–
*S. enterica* R strain KKP 1775	ON832663	+	*Listeria monocytogenes* strain KKP 3270	MT990525	–
*S*. Typhimurium strain KKP 1776	ON461376	+	*Staphylococcus aureus* strain KKP 995	OQ302557	–
*S.* Enteritidis strain KKP 3078	MW034593	+	*Staphylococcus aureus* strain KKP 1082	OQ302555	–

Notes: An abbreviated name is used for each strain of *Salmonella* serovar listed in the table; for example, *S.* Berta means *Salmonella enterica* subsp. *enterica* serovar Berta; *S*. I means *Salmonella enterica* subsp. *enterica*. “R”—rough strain; “H”—primary bacterial host strain; “++”—transparent plaques; “+”—turbid (cloudy) plaques; “–”—no plaques (non-susceptible bacterial strain). Each spot test was performed in triplicate (*n* = 3).

**Table 3:** BLASTn alignment of homologous phage sequences (according to GenBank database) with isolated Salmonella phage KKP_3822.

Phage Name (Complete Genome)	Genome Length [bp]	G+C Content [%]	No. of Genes/Protein	E-Value	Query Coverage [%]	Percent Identity [%]	Phage Family/ Genus	Genome	Country of Isolation	Isolation Source	Lab Host	GenBank Accession No.
Salmonella Phage KKP_3822												
Salmonella phage MET_P1_137_112	112,278	40.0	185/160	0.0	92	96.97	*Demerecviridae/Epseptimavirus*	linear dsDNA	Turkey	wastewater	ND	OQ383621.1
Salmonella phage rutana	114,544	40.0	166/166	0.0	90	98.53	*Demerecviridae/Epseptimavirus*	linear dsDNA	Denmark	wastewater	*S.* Enteritidis PT1	MT074468.1
Salmonella phage beppo	111,322	40.0	166/166	0.0	88	98.30	*Demerecviridae/Epseptimavirus*	linear dsDNA	Denmark	wastewater	*S.* Enteritidis PT1	MT074455.1
Escherichia coli phage Shin27 DNA	112,034	40.0	191/162	0.0	93	97.61	*Demerecviridae/Epseptimavirus*	linear dsDNA	Japan	sewage	ND	LC788709.1
Salmonella phage KKP 3953	115,522	40.0	179/179	0.0	87	97.48	*Demerecviridae/Epseptimavirus*	linear dsDNA	Poland	wastewater	*S. enterica* subsp. *enterica* serowar 6,8:1,v:- strain KKP 1008	OR067837.1
Salmonella phage KKP_3831	113,006	39.5	171/160	0.0	85	97.59	*Demerecviridae/Epseptimavirus*	linear dsDNA	Poland	wastewater	*S. enterica* subsp. *enterica* serovar 6,8:l,v:- strain KKP 998	OQ674103.1
Salmonella phage bux	112,486	40.0	160/160	0.0	87	97.49	*Demerecviridae/Epseptimavirus*	linear dsDNA	Denmark	wastewater	*S.* Enteritidis PT1	MT074460.1
Salmonella phage vaffelhjerte	110,831	40.0	160/160	0.0	88	97.30	*Demerecviridae/Epseptimavirus*	linear dsDNA	Denmark	wastewater	*S.* Enteritidis PT1	MT074452.1
Salmonella phage rokbiter	112,006	40.0	192/163	0.0	90	97.22	*Demerecviridae/Epseptimavirus*	linear dsDNA	Denmark	wastewater	*S.* Enteritidis PT1	NC_048868.1
Escherichia virus vB_Eco_mar003J3	115,471	39.8	189/163	0.0	85	97.11	*Demerecviridae/Epseptimavirus*	linear dsDNA	USA	sea water	ND	NC_048203.1
Salmonella phage atrejo	114,059	40.0	196/167	0.0	90	96.57	*Demerecviridae/Epseptimavirus*	linear dsDNA	Denmark	wastewater	*S.* Enteritidis PT1	NC_048872.1
Salmonella phage gmork	113,259	40.0	167/167	0.0	90	96.44	*Demerecviridae/Epseptimavirus*	linear dsDNA	Denmark	wastewater	*S.* Enteritidis PT1	MT074463.1
Salmonella phage RA40	111,611	40.0	190/162	0.0	90	96.10	*Demerecviridae/Epseptimavirus*	linear dsDNA	United Kingdom	sewage	*S*. Typhimurium	OR242313.1
Salmonella phage vB_SenS-3	119,586	40.0	230/229	0.0	90	95.55	*Demerecviridae/Epseptimavirus*	linear dsDNA	Poland	sewage	*S. enterica*	MT004791.1
Salmonella phage polluks	111,599	40.0	162/162	0.0	89	95.48	*Demerecviridae/Epseptimavirus*	linear dsDNA	Denmark	wastewater	*S.* Enteritidis PT1	MT074456.1

ND—no data.

## Data Availability

The original contributions presented in the study are included in the article. Further inquiries can be directed to the corresponding authors.
